# Retrospective cohort study of trifluridine/tipiracil (TAS-102) plus bevacizumab versus trifluridine/tipiracil monotherapy for metastatic colorectal cancer

**DOI:** 10.1186/s12885-019-6475-6

**Published:** 2019-12-27

**Authors:** Daisuke Kotani, Yasutoshi Kuboki, Satoshi Horasawa, Asumi Kaneko, Yoshiaki Nakamura, Akihito Kawazoe, Hideaki Bando, Hiroya Taniguchi, Kohei Shitara, Takashi Kojima, Akihito Tsuji, Takayuki Yoshino

**Affiliations:** 1 Department of Gastrointestinal Oncology, National Cancer Center Hospital East, Kashiwa, Japan; 2 Department of Experimental Therapeutics, National Cancer Center Hospital East, 6-5-1 Kashiwanoha, Kashiwa, Chiba 277-8577 Japan; 3 Translational Research Support Section, Translational Research Management Division, Clinical Research Support Office, National Cancer Center Hospital East, Kashiwa, Japan; 4 Department of Pharmacy, National Cancer Center Hospital East, Kashiwa, Japan; 5 Department of Clinical Oncology, Aichi Cancer Center Hospital, Nagoya, Japan; 6 Department of Medical Oncology, Graduated School of Medicine, Kagawa University, Takamatsu, Japan

**Keywords:** Trifluridine/tipiracil plus bevacizumab, TAS-102, mCRC, Lonsurf

## Abstract

**Background:**

A previous phase I/II C-TASK FORCE study of trifluridine/tipiracil plus bevacizumab for patients with heavily pretreated metastatic colorectal cancer (mCRC) showed promising activity with an acceptable toxicity profile. This retrospective study aimed to investigate the safety and efficacy of trifluridine/tipiracil plus bevacizumab compared with trifluridine/tipiracil monotherapy in patients with heavily pretreated mCRC in clinical settings.

**Methods:**

Records of patients with mCRC refractory to standard therapies who initiated trifluridine/tipiracil plus bevacizumab from January 2016 to March 2018 or trifluridine/tipiracil monotherapy from June 2014 to December 2015 were retrospectively reviewed at our institution.

**Results:**

Totally, 60 patients received trifluridine/tipiracil plus bevacizumab and 66 received trifluridine/tipiracil monotherapy. All patients had previously received standard chemotherapy. Median progression-free survival (PFS) was 3.7 months [95% confidence interval (CI), 2.3–5.1] in the trifluridine/tipiracil plus bevacizumab group and 2.2 months (95% CI, 1.8–2.6) in the trifluridine/tipiracil monotherapy group [hazards ratio (HR) 0.69; 95% CI 0.48–0.99]. PFS rate at 16 weeks was 46.6% for the trifluridine/tipiracil plus bevacizumab group and 33.9% for the trifluridine/tipiracil monotherapy group. Although a relatively higher incidence of grade ≥ 3 neutropenia was observed in the trifluridine/tipiracil plus bevacizumab group than that in the other group (50.0% vs. 40.9%, *p* = 0.371), the incidence of febrile neutropenia was not high (3.3% vs. 7.8%, *p* = 0.444).

**Conclusions:**

In real-world settings, trifluridine/tipiracil plus bevacizumab prolonged PFS and helped achieve higher 16-week PFS rate compared with trifluridine/tipiracil monotherapy in patients with heavily pretreated mCRC with manageable toxicities.

**Trial registration:**

Retrospectively registered.

## Background

Colorectal cancer (CRC) is the second leading cause of cancer-related death worldwide [1]. The development of combination chemotherapy regimens comprising cytotoxic agents (e.g., fluoropyrimidine, oxaliplatin, and irinotecan) and molecular targeted therapies (e.g., bevacizumab, ramucirumab, ziv-aflibercept, cetuximab, panitumumab, and regorafenib) have increased the survival of patients with metastatic CRC (mCRC) by approximately 30 months [2–7].

Trifluridine/tipiracil (TAS-102) is a novel, oral combination comprising the thymidine-based nucleoside analog trifluridine and tipiracil hydrochloride at a molar ratio of 1:0.5. Trifluridine is incorporated into DNA after phosphorylation by thymidine kinase 1 (TK1). We previously reported results from a randomized phase 2 study of trifluridine/tipiracil (J003–10040030), and this therapy was first approved in Japan in 2014 [8]. More recently, the international phase 3 RECOURSE trial has demonstrated a more significant overall survival (OS) benefit of trifluridine/tipiracil compared with placebo, with acceptable toxicity, in patients with refractory mCRC [7]. In addition, the Asian phase 3 TERRA trial has reported the survival benefit and safety of trifluridine/tipiracil in Asian population [9]^.^ Based on these findings, trifluridine/tipiracil has been approved by many countries and regions including the US Food and Drug Administration and European Medicines Agency.

The combination of trifluridine/tipiracil plus bevacizumab has been demonstrated to have synergistic activity in a xenograft model of human CRC [10]. We have reported results from the phase 1/2 C-TASK FORCE showing a promising activity of the aforementioned combination in patients with pretreated mCRC. The primary endpoint of 16-week progression-free survival (PFS) was 42.9% [80% confidence interval (CI), 27.8–59.0]. The most common grade ≥ 3 adverse events were neutropenia (72%), leucopenia (44%), anemia (16%), febrile neutropenia (16%), and thrombocytopenia (12%) [11]. Managing the higher incidence of hematological toxicities is crucial to reduce the risks of serious treatment-related adverse events and maximize the efficacy of trifluridine/tipiracil plus bevacizumab treatment. Furthermore, the non-comparative phase 2 TASCO1 study evaluated the efficacy and safety of trifluridine/tipiracil plus bevacizumab and capecitabine plus bevacizumab and provided evidence demonstrating the efficacy of trifluridine/tipiracil plus bevacizumab in patients with untreated mCRC who were not eligible for standard first-line intensive therapy. The primary endpoint of PFS was 9.2 months in the trifluridine/tipiracil plus bevacizumab group and 7.8 months in the capecitabine plus bevacizumab group [12].

Although trifluridine/tipiracil plus bevacizumab is a promising regimen for mCRC patients, little is known about the survival benefit and safety profile of the combination compared with trifluridine/tipiracil monotherapy. Therefore, the aim of this study was to investigate efficacy and safety of trifluridine/tipiracil plus bevacizumab compared with trifluridine/tipiracil monotherapy in the clinical practice.

## Methods

### Study design and patients

Clinical data of patients with mCRC who received trifluridine/tipiracil plus bevacizumab or trifluridine/tipiracil monotherapy at the National Cancer Center Hospital East was retrospectively collected. Study protocol was approved by the institutional review board. Informed consent requirement was waived due to the study’s observational retrospective design, with an opt-out opportunity provided at the institution’s website. Patient follow-up was performed until December 2018.

Eligibility criteria were as follows: histologically confirmed colorectal adenocarcinoma; no prior treatment with regorafenib; refractory or intolerant to fluoropyrimidine, oxaliplatin, and irinotecan, regardless of angiogenesis inhibitors or anti-EGFR antibody (if *RAS* wild-type); Eastern Cooperative Oncology Group performance status (ECOG PS) 0 to 2; adequate organ function; concurrent treatment with trifluridine/tipiracil plus bevacizumab from January 2016 to March 2018 or trifluridine/tipiracil monotherapy from June 2014 to December 2015.

### Study procedures

Trifluridine/tipiracil plus bevacizumab regimen consisted of trifluridine/tipiracil 35 mg/m^2^ of body surface area, given orally twice a day on days 1–5 and 8–12 in a 28-day cycle, and bevacizumab 5 mg/kg of bodyweight, administered by intravenous infusion every 2 weeks. Trifluridine/tipiracil monotherapy consisted of trifluridine/tipiracil 35 mg/m^2^ of body surface area, given orally twice a day on days 1–5 and 8–12 in a 28-day cycle.

The following baseline characteristics were collected for each patient: age, gender, ECOG PS, primary tumor location, history of primary resection, number of metastatic organs, time from first-line chemotherapy start, time from prior bevacizumab, prior chemotherapy agents, *RAS* status (*KRAS* exons 2, 3, and 4 and *NRAS* exons 2, 3, and 4), *BRAF* V600E mutation status, and microsatellite instability (MSI) status, if available.

### Outcomes

Efficacy endpoints included PFS, defined as time from study treatment initiation to disease progression or death due to any cause; OS, defined as time from study treatment start to death from any cause; overall response rate (ORR), defined as proportion of patients with a complete or partial response to study treatment; disease control rate (DCR), defined as proportion of patients with a complete or partial response plus stable disease lasting more than 6 weeks from study treatment start. Tumor response was assessed by investigators using the Response Evaluation Criteria in Solid Tumors version 1.1. Adverse events were evaluated using the Common Terminology Criteria for Adverse Events version 4.03.

### Statistical analysis

PFS and OS were compared between treatment groups using log-rank test with a two-sided significance level of *p* = 0.05. Hazard ratio (HR) and corresponding 95% CI were determined using a Cox proportional hazard model. Survival curves were generated using Kaplan-Meier estimates. Univariate and multivariate analyses were performed to evaluate the impact of trifluridine/tipiracil plus bevacizumab or trifluridine/tipiracil monotherapy treatments. Regression analysis covariates included treatment group, age, gender, ECOG PS, primary tumor location, *RAS* status, time from first-line chemotherapy start, and time from prior bevacizumab. Multivariate Cox analysis was employed using forward stepwise regression. Enter and remove limits were *p* = 0.05 and *p* = 0.20, respectively. Confounding variables considered in multivariate analysis included age (< 65 years old vs. ≥65 years old), gender (male vs. female), ECOG PS (0 vs. ≥1), primary tumor location (right vs. left), *RAS* status (wild-type vs. mutant), time from first-line chemotherapy start (≥18 months vs. < 18 months), and time from prior bevacizumab (≤1 month vs. > 1 month or no prior bevacizumab). ORR, DCR, and safety analyses between treatment groups were performed using Fisher’s exact test. Follow-up time was defined as time from study treatment start until the last follow-up date for censored cases. Statistical analyses were performed using IBM SPSS statistics version 22.0 (IBM Corp, Armonk, NY), and two-sided *p* < 0.05 was considered statistically significant.

## Results

### Patients

Sixty patients received trifluridine/tipiracil plus bevacizumab and 66 patients received trifluridine/tipiracil monotherapy. Patient demographics and baseline characteristics are shown in Table 1. All patients had previously received fluoropyrimidine, oxaliplatin, and irinotecan. Most of the patients in each group had a history of treatment with angiogenesis inhibitors, including bevacizumab, ramucirumab, or ziv-aflibercept. Approximately half of patients had *RAS* wild-type tumors, and no patient had MSI-high tumor. *BRAF* V600E mutation was detected in one patient (1.7%) in the trifluridine/tipiracil plus bevacizumab group and in four patients (6.1%) in the trifluridine/tipiracil monotherapy group. Patients with left-sided primary tumors were dominant in both groups and comprised 81.7% of the trifluridine/tipiracil plus bevacizumab group and 84.8% of the trifluridine/tipiracil monotherapy group. Median interval from study treatment start to first computed tomography evaluation was 1.8 months in both groups. Median follow-up was 7.1 months in the trifluridine/tipiracil plus bevacizumab groups and 7.2 months in the trifluridine/tipiracil monotherapy group. After study treatment discontinuation, 41.7% of patients in the trifluridine/tipiracil plus bevacizumab groups and 48.5% of patients in the trifluridine/tipiracil monotherapy group received subsequent antitumor therapy including regorafenib (31.7 vs. 39.4%), clinical trial therapy (6.7 vs. 4.5%), and cytotoxic chemotherapy (3.3 vs. 6.0%).

**Table 1:** Patient characteristics

		Trifluridine/tipiracil plus bevacizumab group		Trifluridine/tipiracil monotherapy group	
		*N* = 60	%	*N* = 66	%
Age	Median (range)	60 (23–79)		65 (30–80)	
	≥65 years old	19	31.7	34	51.5
Gender	Male	35	58.3	42	63.6
ECOG PS	0	35	58.3	42	63.6
	1	24	40.0	21	31.8
	2	1	1.7	3	4.5
Primary location	Right	11	18.3	10	15.2
	Left	49	81.7	56	84.8
Number of metastatic organs	1	6	10.0	13	19.7
	2	26	43.3	39	59.1
	≥3	28	46.7	14	21.2
Time from start of first-line chemotherapy	< 18 months	22	36.7	23	34.8
	≥18 months	38	63.3	43	65.2
Time from prior bevacizumab	≤1 month	34	56.7	33	50.0
	> 1 month or no prior bevacizumab	26	43.3	33	50.0
Number of prior regimens	1	4	6.7	2	3.0
	2	29	48.3	33	50.0
	3	15	25.0	16	24.2
	≥4	12	20.0	15	22.7
Prior chemotherapy agents	Fluoropyrimidine	60	100	66	100
	Irinotecan	60	100	66	100
	Oxaliplatin	60	100	66	100
	Angiogenesis inhibitors	58	96.7	61	92.4
	Anti-EGFR antibodies	27	45.0	27	40.9
*RAS* status	Wild-type	28	46.7	30	45.5
	Mutant	32	53.3	36	54.5
*BRAF* status	Wild-type	52	86.7	52	78.8
	V600E mutant	1	1.7	4	6.1
	Non-V600E mutant	2	3.3	0	0
	Unknown	5	8.3	10	15.2
MSI status	MSS	53	88.3	51	77.3
	Unknown	7	11.7	15	22.7

*ECOG PS* Eastern Cooperative Oncology Group performance status, *EGFR* epidermal growth factor receptor, *MSS* microsatellite stable

### Efficacy

**Fig. 1:**
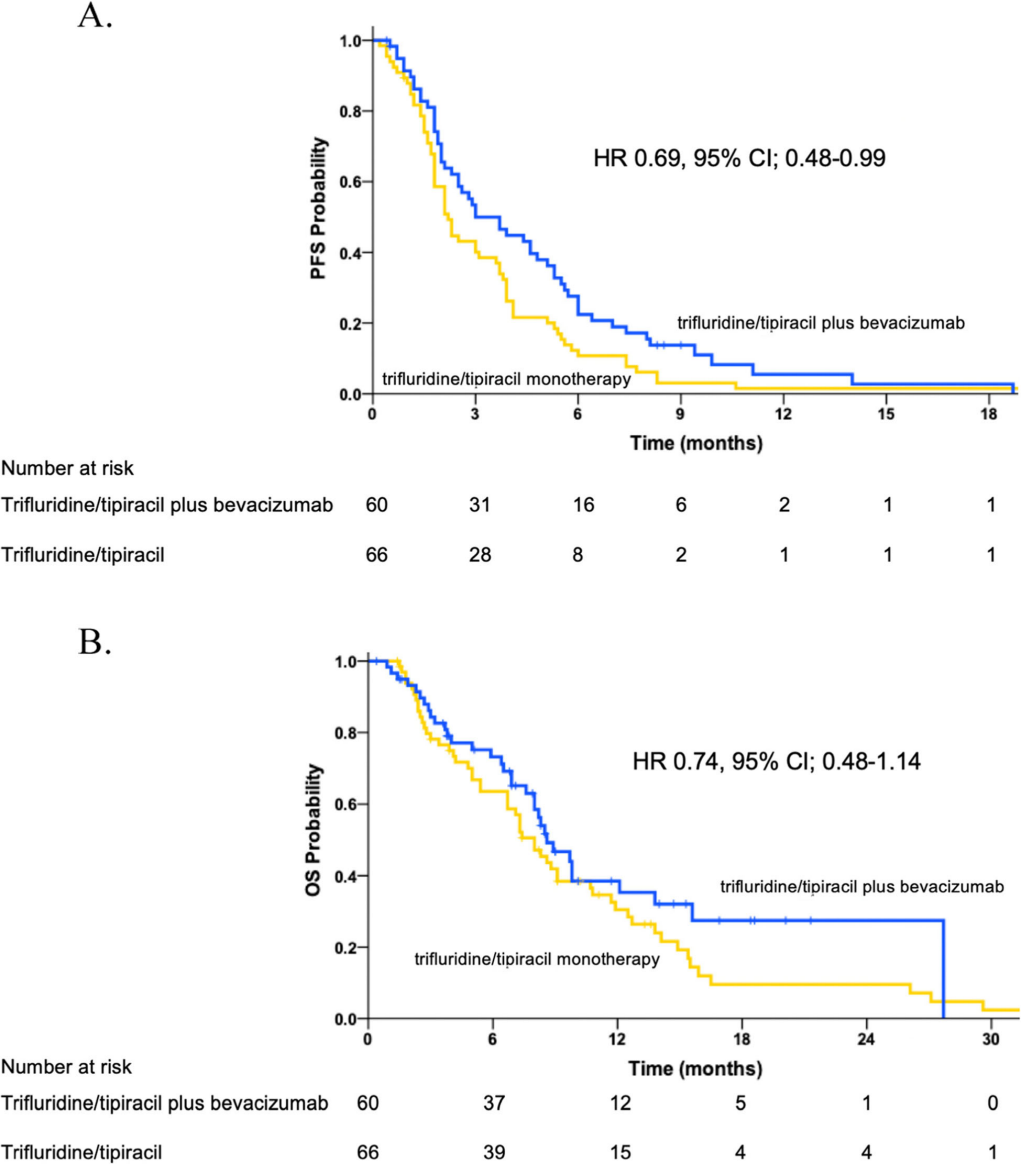
**a** Kaplan–Meier plots for PFS. **b** Kaplan–Meier plots for OS

Patients in the trifluridine/tipiracil plus bevacizumab group had significantly longer PFS compared with those in the trifluridine/tipiracil monotherapy group (HR 0.69; 95% CI 0.48–0.99; log-rank *p* = 0.037). Median PFS was 3.7 months (95% CI 2.3–5.1 months) in the trifluridine/tipiracil plus bevacizumab group and 2.2 months (95% CI 1.8–2.6 months) in the trifluridine/tipiracil monotherapy group (Fig. 1a). PFS rate at 16 weeks was 46.6 and 33.9%, respectively. In multivariate analysis, similar PFS was observed between groups (adjusted HR 0.62; 95% CI 0.42–0.90, *p* = 0.01). Subgroup analyses also showed a PFS benefit for trifluridine/tipiracil plus bevacizumab compared with trifluridine/tipiracil monotherapy for all parameters except ECOG PS (Table 2).

**Table 2:** PFS and OS subgroup analyses

			PFS			OS		
		N	HR	95% CI	Interaction p	HR	95% CI	Interaction p
All patients		126	0.69	0.48–0.99		0.74	0.48–1.14	
Age	< 65 years old	73	0.53	0.31–0.88	0.542	0.85	0.48–1.50	0.300
	≥65 years old	53	0.74	0.41–1.32		0.49	0.23–1.08	
Gender	Male	77	0.72	0.45–1.15	0.560	0.98	0.57–1.70	0.162
	Female	49	0.61	0.34–1.09		0.51	0.25–1.04	
ECOG PS	0	77	0.50	0.31–0.80	0.009	0.54	0.30–0.99	0.033
	≥1	49	1.42	0.79–2.55		1.46	0.76–2.82	
Primary location	Left	105	0.65	0.44–0.98	0.758	0.72	0.44–1.16	0.986
	Right	21	0.64	0.26–1.59		0.62	0.21–1.80	
Time from start of first-line chemotherapy	≥18 months	81	0.71	0.45–1.11	0.359	0.66	0.38–1.16	0.635
	< 18 months	45	0.67	0.36–1.25		0.78	0.39–1.55	
Time from prior bevacizumab	≤1 month	67	0.48	0.28–0.82	0.165	0.64	0.37–1.12	0.975
	> 1 month or no prior bevacizumab	59	0.77	0.45–1.33		0.73	0.36–1.45	
*RAS* status	Wild-type	58	0.87	0.50–1.49	0.147	0.67	0.35–1.28	0.580
	Mutant	68	0.52	0.31–0.87		0.79	0.44–1.41	
History of bevacizumab	yes	118	0.75	0.51–1.09	0.47	0.70	0.45–1.09	0.88
	no	8	0.50	0.09–2.67		3.30	0.29–37.7	

*CI* confidence interval, *ECOG PS* Eastern Cooperative Oncology Group performance status, *HR* hazard ratio, *OS* overall survival, *PFS* progression-free survival

Median OS was 8.6 months (95% CI 6.9–10.3 months) for trifluridine/tipiracil plus bevacizumab and 8.0 months (95% CI 6.6–9.4 months) for trifluridine/tipiracil monotherapy (Fig. 1b). In multivariate analysis, an OS benefit was also observed for trifluridine/tipiracil plus bevacizumab compared with trifluridine/tipiracil monotherapy, but without statistical significance (HR 0.74; 95% CI 0.48–1.14; log-rank *p* = 0.164). Similarly to PFS, longer OS was observed for all subgroups, except ECOG PS.

Three patients in the trifluridine/tipiracil plus bevacizumab group and one patient in the trifluridine/tipiracil monotherapy group had partial response, resulting in a 5.0 and 1.5% ORR for each group, respectively (*p* = 0.35). Disease control was achieved in 32 patients (53.3%) in the trifluridine/tipiracil plus bevacizumab group and in 30 patients (45.5%) in the trifluridine/tipiracil monotherapy group (*p* = 0.48) (Table 3). Additionally, proportion of patients with 6 months or longer of disease control were significantly higher in the trifluridine/tipiracil plus bevacizumab group than the trifluridine/tipiracil monotherapy group (26.7% vs. 12.1%, *p* = 0.04).

**Table 3:** Overall response

Best response	Trifluridine/tipiracil plus bevacizumab group		Trifluridine/tipiracil monotherapy group		*P*
	*N* = 60	%	*N* = 66	%	
PR	3	5.0	1	1.5	
SD	29	48.3	29	43.9	
PD	25	41.7	34	51.5	
NE	3	5.0	2	3.0	
ORR	3	5.0	1	1.5	0.346
DCR	32	53.3	30	45.5	0.476

*DCR* disease control rate, *NE* not evaluated, *ORR* overall response rate, *PD* progressive disease, *PR* partial response, *SD* stable disease

### Safety

All patients initially received full-dose trifluridine/tipiracil and bevacizumab or trifluridine/tipiracil monotherapy. Any cycle delay for ≥4 days was registered in 37 (61.7%) and 27 (40.9%) patients in the trifluridine/tipiracil plus bevacizumab group and trifluridine/tipiracil monotherapy groups (*p* = 0.02), and trifluridine/tipiracil dose reductions were required in 10 (16.7%) and 15 (22.7%) patients (*p* = 0.50), respectively.

Adverse events are summarized in Table 4. Overall, grade ≥ 3 adverse events were more frequent in the trifluridine/tipiracil plus bevacizumab than in the trifluridine/tipiracil monotherapy group (*n* = 41, 68.3% vs. *n* = 36, 54.5%; *p* = 0.14). Incidence of grade ≥ 3 neutropenia was slightly higher in the trifluridine/tipiracil plus bevacizumab group than in the trifluridine/tipiracil monotherapy group, although this difference was not statistically significant (50.0% vs. 40.9%; *p* = 0.37). In contrast, no increased incidence of febrile neutropenia was observed in the trifluridine/tipiracil plus bevacizumab compared with the trifluridine/tipiracil monotherapy group (3.3% vs. 7.8%; *p* = 0.444). Ten patients (16.7%) in the trifluridine/tipiracil plus bevacizumab group and four patients (6.1%) in the trifluridine/tipiracil monotherapy group received granulocyte colony-stimulating factor (G-CSF), with no G-CSF prophylaxis use in the both groups. Any grade proteinuria (41.7% vs. 13.6%; *p* < 0.01) and hypertension (38.3% vs. 16.7%; *p* < 0.01), potentially associated with bevacizumab, were more common in the trifluridine/tipiracil plus bevacizumab group. Emergency hospital admission was required for 15 (25.0%) and 19 (28.8%) patients in the trifluridine/tipiracil plus bevacizumab and trifluridine/tipiracil monotherapy groups, respectively. No treatment-related deaths occurred in the both groups.

**Table 4:** Adverse events

	Trifluridine/tipiracil plus bevacizumab group (*N* = 60)			Trifluridine/tipiracil monotherapy group (*N* = 66)		
	Any grade (%)	Grade 3 (%)	Grade 4 (%)	Any grade (%)	Grade 3 (%)	Grade 4 (%)
Hematological						
Neutropenia	41 (68.3)	25 (41.7)	5 (8.3)	47 (71.2)	19 (28.8)	8 (12.1)
Leucopenia	49 (81.7)	23 (38.3)	0 (0)	47 (71.2)	17 (25.8)	2 (3.0)
Anemia	52 (86.7)	8 (13.3)	1 (1.7)	60 (90.9)	12 (18.2)	2 (3.0)
Thrombocytopenia	26 (43.3)	1 (1.7)	1 (1.7)	24 (36.4)	2 (3.0)	0 (0)
Non-hematological						
Proteinuria	25 (41.7)	4 (6.7)	0 (0)	9 (13.6)	1 (1.5)	0 (0)
Hypertension	23 (38.3)	4 (6.7)	0 (0)	11 (16.7)	0 (0)	0 (0)
Febrile neutropenia	2 (3.3)	2 (3.3)	0 (0)	5 (7.8)	5 (7.8)	0 (0)
Gastrointestinal perforation	2 (3.3)	2 (2.9)	0 (0)	1 (1.5)	1 (1.5)	0 (0)
Fatigue	30 (50.0)	0 (0)	0 (0)	25 (37.9)	0 (0)	0 (0)
Anorexia	25 (41.7)	0 (0)	0 (0)	27 (40.9)	1 (1.5)	0 (0)
Nausea	10 (16.7)	0 (0)	0 (0)	15 (22.7)	0 (0)	0 (0)
Diarrhea	5 (8.3)	0 (0)	0 (0)	8 (12.1)	0 (0)	0 (0)
Vomiting	2 (3.3)	0 (0)	0 (0)	4 (6.1)	0 (0)	0 (0)

## Discussion

Although the clinical evidence for trifluridine/tipiracil plus bevacizumab in pretreated mCRC patients only comes from a phase 1/2 C-TASK FORCE trial with a single-arm design, the present study showed clinical benefit with manageable toxicities for this combination in the real-world setting.

The PFS improvement associated with trifluridine/tipiracil plus bevacizumab can be regarded as clinically meaningful in this patient population. Considering the median PFS difference between trifluridine/tipiracil and placebo of 0.3 and 0.2 months reported in RECOURSE and TERRA trials, respectively, the absolute 1.5-month median PFS improvement observed in this study is clinically meaningful in salvage setting. Of note, a higher 16-week PFS rate was observed with trifluridine/tipiracil plus bevacizumab compared with trifluridine/tipiracil monotherapy (46.6 vs. 33.9%), consistent with the primary endpoint of the C-TASK FORCE trial. Furthermore, although limited efficacy was reported in the C-TASK FORCE trial for trifluridine/tipiracil plus bevacizumab in patients with mutant *RAS*, a consistent benefit of the combination was observed in subgroup analyses in this study, irrespectively of *RAS* status. Although not statistically significant, a 0.74 HR for OS was observed in favor of trifluridine/tipiracil plus bevacizumab in this study. Fewer OS events in the trifluridine/tipiracil plus bevacizumab group due to limited follow-up may have failed to show statistical difference between both groups. In subgroup analyses, patients with ECOG PS 0 especially benefited from trifluridine/tipiracil plus bevacizumab in both PFS and OS. Although no clear benefit of trifluridine/tipiracil plus bevacizumab were observed in patients with ≥ ECOG PS 1, it is not sufficient reason to refrain from using trifluridine/tipiracil plus bevacizumab in those patients, considering retrospective nature of this study.

Trifluridine/tipiracil plus bevacizumab toxicities were well tolerated in the clinical practice setting. Frequency of grade ≥ 3 neutropenia in the trifluridine/tipiracil plus bevacizumab group in this study was lower than in the C-TASK FORCE trial (50.0 vs. 72%). Along with a relatively lower frequency of grade ≥ 3 neutropenia, G-CSF was used in 16.8% of patients in the trifluridine/tipiracil plus bevacizumab group in this study compared with 28% of patients in the C-TASK FORCE trial. However, considering the common occurrence of grade ≥ 3 neutropenia, the higher proportion of cycle initiation delay in the trifluridine/tipiracil plus bevacizumab group, and the patient population in salvage setting, appropriate supportive intervention with G-CSF or temporary dose interruptions were still important for safety management with trifluridine/tipiracil plus bevacizumab. As bevacizumab – related toxicities, incidence of any grade proteinuria and hypertension were significantly higher in the trifluridine/tipiracil plus bevacizumab group. In the salvage line setting, since almost all patients have received one or more prior angiogenesis inhibitor including bevacizumab, these bevacizumab – related toxicities should be monitored carefully. Notably, such safety management was not associated with an increase in emergency hospital admissions with trifluridine/tipiracil plus bevacizumab, and the incidence of serious adverse events in this study was similar to the observed in RECOURSE and C-TASK FORCE trials and in clinical practice, as previously reported from our institution [13].

Efficacy and safety of trifluridine/tipiracil plus bevacizumab is also being investigated in prospective clinical trials. The phase 2 JFMC51–1702-C7 study (UMIN000030077) will validate the use of trifluridine/tipiracil plus bevacizumab in pretreated mCRC patients. Furthermore, this combination is an effective (ORR 40.5% and DCR 86.5%) and well-tolerated first-line treatment regimen for elderly patients (KSCC1602; UMIN000025241) [14]. More recently, a randomized Danish study of 80 patients with heavily pretreated mCRC reported a PFS extension with trifluridine/tipiracil plus bevacizumab compared with trifluridine/tipiracil monotherapy [15]. Based on the above-mentioned findings, several phase 2 or 3 studies are in place, including studies investigating this regimen as post-induction chemotherapy maintenance (ALEXANDRIA; NCT02654639) and as second-line treatment for mCRC patients who failed first-line oxaliplatin-based chemotherapy versus FOLFIRI (or S-1 plus irinotecan) plus bevacizumab (TRUSTY; JapicCTI-173,618) [16].

This study has several limitations. Firstly, as previously noted, it was a non-randomized retrospective study in a single institution with a limited sample size. Secondly, no patients received prior regorafenib. However, the C-TASK FORCE trial also did not allow prior regorafenib and the study population was very similar to the Japanese subset of the RECOURSE trial. Finally, all patients enrolled in this study were Japanese. The absence of ethnic differences in the RECOURSE and TERRA trials could enable results to be applied to all patients, regardless of race.

## Conclusions

In conclusion, in the present study trifluridine/tipiracil plus bevacizumab was shown to have superior clinical activity compared with trifluridine/tipiracil monotherapy in patients with heavily pretreated mCRC. Additionally, similarly to trifluridine/tipiracil monotherapy, toxicities observed with the combination were manageable in the real-world setting.

## Data Availability

Not applicable.

## Abbreviations

CI: Confidence interval
CRC: Colorectal cancer
DCR: Disease control rate
ECOG PS: Eastern Cooperative Oncology Group performance status
G-CSF: Granulocyte colony-stimulating factor
HR: Hazards ratio
mCRC: Metastatic colorectal cancer
MSI: Microsatellite instability
ORR: Overall response rate
OS: Overall survival
PFS: Progression-free survival
TK1: Thymidine kinase 1
